# Advancing 6-bromo-7-[^11^C]methylpurine to clinical use: improved regioselective radiosynthesis, non-clinical toxicity data and human dosimetry estimates

**DOI:** 10.1186/s41181-024-00265-z

**Published:** 2024-04-29

**Authors:** Severin Mairinger, Matthias Jackwerth, Ondřej Soukup, Matthias Blaickner, Clemens Decristoforo, Lukas Nics, Jens Pahnke, Marcus Hacker, Markus Zeitlinger, Oliver Langer

**Affiliations:** 1 Department of Clinical Pharmacology, Medical University of Vienna, Vienna, Austria; 2 Department of Biomedical Imaging and Image-Guided Therapy, Medical University of Vienna, Vienna, Austria; 3 Biomedical Research Center, University Hospital Hradec Kralove, Hradec Kralove, Czech Republic; 4 Department Computer Science, University of Applied Sciences Technikum Wien, Vienna, Austria; 5 Department of Nuclear Medicine, Medical University Innsbruck, Innsbruck, Austria; 6 Translational Neurodegeneration Research and Neuropathology Lab, Department of Clinical Medicine (KlinMed), Medical Faculty, University of Oslo, Oslo, Norway; 7 Section of Neuropathology Research, Department of Pathology, Clinics for Laboratory Medicine (KLM), Oslo University Hospital, Oslo, Norway; 8 Institute of Nutritional Medicine (INUM) and Lübeck Institute of Dermatology (LIED), University of Lübeck and University Medical Center Schleswig-Holstein, Lübeck, Germany; 9 Department of Pharmacology, Faculty of Medicine, University of Latvia, Rīga, Latvia; 10 School of Neurobiology, Biochemistry and Biophysics, The Georg S. Wise Faculty of Life Sciences, Tel Aviv University, Tel Aviv, Israel

**Keywords:** 6-Bromo-7-[^11^C]methylpurine, Investigational medicinal product dossier, Multidrug resistance-associated protein 1, PET, Regioselective *N*‑alkylation

## Abstract

**Background:**

6-Bromo-7-[^11^C]methylpurine ([^11^C]BMP) is a radiotracer for positron emission tomography (PET) to measure multidrug resistance-associated protein 1 (MRP1) transport activity in different tissues. Previously reported radiosyntheses of [^11^C]BMP afforded a mixture of 7- and 9-[^11^C]methyl regioisomers. To prepare for clinical use, we here report an improved regioselective radiosynthesis of [^11^C]BMP, the results of a non-clinical toxicity study as well as human dosimetry estimates based on mouse PET data.

**Results:**

[^11^C]BMP was synthesised by regioselective *N*^*7*^-methylation of 6-bromo-7H-purine (prepared under good manufacturing practice) with [^11^C]methyl triflate in presence of 2,2,6,6-tetramethylpiperidine magnesium chloride in a TRACERlab™ FX2 C synthesis module. [^11^C]BMP was obtained within a total synthesis time of approximately 43 min in a decay-corrected radiochemical yield of 20.5 ± 5.2%, based on starting [^11^C]methyl iodide, with a radiochemical purity > 99% and a molar activity at end of synthesis of 197 ± 130 GBq/μmol (*n* = 28). An extended single-dose toxicity study conducted in male and female Wistar rats under good laboratory practice after single intravenous (i.v.) administration of unlabelled BMP (2 mg/kg body weight) revealed no test item related adverse effects. Human dosimetry estimates, based on dynamic whole-body PET data in female C57BL/6J mice, suggested that an i.v. injected activity amount of 400 MBq of [^11^C]BMP will deliver an effective dose in the typical range of ^11^C-labelled radiotracers.

**Conclusions:**

[^11^C]BMP can be produced in sufficient amounts and acceptable quality for clinical use. Data from the non-clinical safety evaluation showed no adverse effects and suggested that the administration of [^11^C]BMP will be safe and well tolerated in humans.

**Supplementary Information:**

The online version contains supplementary material available at 10.1186/s41181-024-00265-z.

## Background

Multidrug resistance-associated protein 1 (MRP1), encoded by the *ABCC1* gene, is an adenosine triphosphate-binding cassette (ABC) transporter with a widespread tissue distribution (Cole 2014). It plays an important role in the efflux of drugs and endogenous compounds from cells and has been implicated in the pathophysiology of various diseases, including Alzheimer's disease (Krohn et al. 2011) and chronic respiratory diseases (van der Deen et al. 2006). MRP1 may be involved in the clearance of amyloid-beta (Aβ) peptides across the blood–brain- and blood-cerebrospinal fluid barriers into the blood and has been suggested as a potential therapeutic target in Alzheimer's disease patients to enhance brain Aβ clearance (Krohn et al. 2011). To advance such a treatment approach to humans, methodology for assessing MRP1 activity in the human brain is essential (Pahnke et al. 2014).

6-Bromo-7-[^11^C]methylpurine ([^11^C]BMP) has been developed as a positron emission tomography (PET) radiotracer to measure MRP1 activity in the brain (Okamura et al. 2009b). [^11^C]BMP is a “pro-tracer” which is itself not a substrate of MRP1. It is rapidly conjugated with glutathione by cytosolic glutathione-*S*-transferase enzymes to afford *S*-(6-(7-[^11^C]methylpurinyl))glutathione, which is effluxed from cells by MRP1 (Okamura et al. 2009b). [^11^C]BMP has been used to measure MRP1 activity in the brain and lungs of mice and rats (Krohn et al. 2019; Mairinger et al. 2023, 2020; Okamura et al. 2009b, 2013; Zoufal et al. 2020, 2019), but has not been tested in humans yet.

The published radiosynthesis procedures of [^11^C]BMP comprise a standard methylation reaction of the desmethyl-precursor 6-bromo-7H-purine with [^11^C]methyl iodide ([^11^C]CH_3_I) or [^11^C]methyl triflate ([^11^C]CH_3_OTf) in the presence of potassium carbonate (K_2_CO_3_) (Okamura et al. 2009b; Zoufal et al. 2019). These reactions yield two isomeric products, with the main product having the ^11^C-label at the *N*^*9*^ position of the purine ring. Based on the reaction rate with glutathione, the optimal position for ^11^C-labelling is the *N*^*7*^ position of the 6-bromopurine heterocycle (Okamura et al. 2007, 2009a). Despite recent efforts to enhance the selectivity for [^11^C]methylation at the *N*^7^ over the *N*^9^ position, current methods still produce a mixture of both isomers requiring normal-phase semi-preparative high-performance liquid chromatography (HPLC) for separation (Okamura et al. 2024). The use of normal-phase HPLC and the necessity to evaporate HPLC solvent prior to product formulation may be impractical for the implementation of this approach for clinical routine production.

**Fig. 1:**
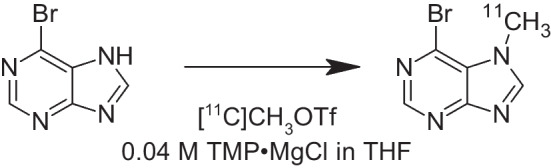
Synthesis of [^11^C]BMP by reaction of 6-bromo-7H-purine with [^11^C]CH_3_OTf in the presence of TMP·MgCl

Here we report a highly reliable and robust [^11^C]BMP radiosynthesis procedure based on regioselective [^11^C]methylation in the *N*^*7*^ position of the purine ring (Chen et al. 2016) for clinical use (Fig. 1). The preparation process including the quality control was developed and validated in order to meet the requirements for preparing an Investigational Medicinal Product Dossier (IMPD) for clinical trial application (Todde et al. 2014). We furthermore report data from 29 consecutive radiosyntheses of [^11^C]BMP, part of which were already used for human application. Moreover, we provide non-clinical safety data that need to be included in the IMPD in the context of a clinical trial application (EMA 2018). These include results from an extended single-dose toxicity study in rats and estimations of human absorbed organ doses and effective doses based on previously published dynamic whole-body PET data obtained in female C57BL/6 J mice after intravenous (i.v.) injection of [^11^C]BMP (Zoufal et al. 2019). This comprehensive approach paves the way for the translation of [^11^C]BMP PET imaging to clinical use.

## Methods

### General

All chemicals were purchased from Sigma-Aldrich Chemie (Schnelldorf, Germany) and Merck KGaA (Darmstadt, Germany) at analytical grade and were used without further purification. 6-Bromo-7H-purine was synthesised with a purity of 99.2% (HPLC) by Mihulka, s.r.o. (Opava-Jaktar, Czech Republic) according to good manufacturing practice (GMP) standards. The identity of 6-bromo-7H-purine was confirmed by ^1^H- and ^13^C-nuclear magnetic resonance (NMR) spectroscopy, high resolution mass spectrometry (HRMS) and attenuated total reflectance (ATR) infrared (IR) spectroscopy. Residual solvents were determined by gas chromatography. Unlabelled 6-bromo-7-methylpurine (BMP, purity (HPLC): 99.42%) and 6-bromo-9-methylpurine (purity (HPLC): 99.96%) were synthesised by Mihulka, s.r.o. in non-GMP quality. The identity of BMP and 6-bromo-9-methylpurine was confirmed by liquid chromatography-mass spectrometry (LC–MS), ^1^H- and ^13^C-NMR and Fourier Transform Infrared (FTIR) spectroscopy. The Hauser base 2,2,6,6-tetramethylpiperidinyl magnesium chloride (TMP·MgCl) was prepared by slowly adding 1 equivalent (eq.) 2,2,6,6-tetramethylpiperidine to 1 eq. butylmagnesium chloride (2.0 M solution in tetrahydrofuran [THF]) under inert atmosphere (Chen et al. 2016). The stirred reaction mixture was heated to reflux for 2 h. The resulting TMP·MgCl solution was further diluted with anhydrous THF to a concentration of 0.04 M. The resulting solution was portioned into 150-µL aliquots under a nitrogen atmosphere.

Radiochemical purity and molar activity of [^11^C]BMP were determined with analytical HPLC using an Agilent 1260 system (Agilent Technologies Österreich GmbH, Vienna, Austria), which was equipped with a quaternary pump (G1311B), a multi wavelength ultraviolet (UV) detector at a wavelength of 274 nm (G1365D), a column oven (G1316A), a manual injector (G1328C) as well as a radio-detector controlled by GINA Star software (Elysia-Raytest, Straubenhardt, Germany). A MultoKrom® 100–5 C18 column (250 × 4.6 mm, 5 µm, 100 Å, CS-Chromatographie Service GmbH, Langerwehe, Germany) was isocratically eluted with a mixture of acetonitrile, water and formic acid (35/65/0.1, *v*/*v*/*v*) at a flow rate of 1 mL/min. Osmolality of the formulated radiotracer solution was measured using a Wescor osmometer Vapro® 5600 (Sanova Medical Systems, Vienna, Austria) and pH was measured using a 913 pH meter (Metrohm Inula GmbH, Vienna, Austria). Gas chromatography was performed using an Intuvo 9000 GC system (Agilent Technologies Österreich GmbH) and a capillary column (DB-624-UI 60 m, 0.32 mm, 1.8 µm) from Agilent Technologies.

### Automated radiosynthesis of [^11^C]BMP

#### Preparation of synthesis module

The automated radiosynthesis was performed in a TRACERlab™ FX2 C synthesis module (GE Healthcare, Uppsala, Sweden). A flow chart of the synthesis module is shown in the Additional file 1: Fig. S1. Before each radiosynthesis, a standardised flow and pressure test was performed on the TRACERlab™ FX2 C system. In the Additional file 1: Fig. S2, a flow chart of the most critical steps in the preparation process for the synthesis of [^11^C]BMP is provided (Todde et al. 2014). The vials V1–V3 of the TRACERlab™ FX2 C system (Additional file 1: Fig. S1) and the corresponding transfer lines via the reactor and the HPLC sample loop were washed once with water and twice with acetone and finally dried with a helium flow of 100 mL/min for at least 5 min. Removal of solvent was assured by visual inspection of all connected tubes. The radiotracer formulation part, including the bulb and product collection vial, was washed with water for injection and ethanol.

The vials were filled with 1 mL of water for injection (V2), 5 mL of aqueous (aq.) 0.9% (*w*/*v*) sodium chloride solution (V4), 1.5 mL of ethanol (V5) and 10 mL of water for injection (V6). The bulb was filled with 80 mL of water for injection, a solid-phase extraction (SPE) cartridge (Oasis HLB Plus Short, Waters, Vienna, Austria) was mounted on the cartridge holder and 8.5 mL of aq. 0.9% sodium chloride solution was filled in the product collection vial. A 25 mL glass vial (TechneVial, DRN 4357, Curium Pharma, Petten, The Netherlands) with a sterile filter (Millex GV, 0.22 µm) and a sterile air ventilation (Millex GV vented, 0.22 µm) was prepared in a laminar air flow cell and connected to the product tubing. Prior to use, the reaction vessel was dried at 110 °C for at least 2 h. Immediately before start of the radiosynthesis, 6-bromo-7H-purine (20–33 nM, 0.6–1.0 mg, 3–5 μmol) was suspended in one pre-prepared aliquot of TMP·MgCl solution in THF (0.04 M, 150 µL, 6 µmol) and the supernatant was transferred with a helium-flushed single-use 1 mL syringe into the reaction vessel, which was then installed in the synthesis module.

#### Process description

[^11^C]Carbon dioxide ([^11^C]CO_2_) was produced in a GE PETtrace 860 cyclotron (General Electric Medical System, Uppsala, Sweden) via the ^14^*N*(p,α)^11^C nuclear reaction by irradiation of a gas target (aluminium) filled with nitrogen + 1% oxygen (Air Liquide, Vienna, Austria). Typical beam currents were 65 μA and the irradiation was stopped as soon as the desired activity level was reached (e.g. approximately 25 min beam time for 80 GBq [^11^C]CO_2_).

The automatic radiosynthesis was then started by delivering [^11^C]CO_2_ to the carbon dioxide trap (4 Å molecular sieve) followed by reduction to [^11^C]methane ([^11^C]CH_4_) over a nickel catalyst with hydrogen at 400 °C. The [^11^C]CH_4_ was trapped on a PorapakQ column at − 75 °C and was further processed to [^11^C]CH_3_I using the standard gas phase method (Larsen et al. 1997). Subsequently, [^11^C]CH_3_I was released and passed through a column filled with silver-triflate-impregnated graphitised carbon heated to 200 °C (Jewett 1992) to generate [^11^C]CH_3_OTf on-line, which was bubbled into the reaction vessel containing 6-bromo-7H-purine suspended in TMP·MgCl/THF solution at 25 °C. After the end of activity transfer, the reaction mixture was heated for 2 min at 70 °C. After cooling (35 °C) and dilution with 1 mL of water, the reaction mixture was injected into the built-in HPLC system. A MultoKrom® 100–5 C18 HPLC column (250 × 10 mm, CS-Chromatographie Service GmbH) was eluted with acetonitrile/water/formic acid (3/97/0.1, *v*/*v*/*v*) at a flow rate of 5 mL/min for 6 min followed by manually switching the mobile phase to acetonitrile/water/formic acid (15/85/0.1, *v*/*v*/*v*). The HPLC eluate was monitored in series for radioactivity and UV absorption at a wavelength of 254 nm. Using this system, 6-bromo-7H-purine and [^11^C]BMP eluted with retention times of approximately 15 min and 17 min, respectively. The product fraction was diluted with water (80 mL) and pushed through the SPE cartridge. The cartridge was washed with 10 mL of water and the product was eluted with 1.5 mL of ethanol into the product collection vial containing 8.5 mL of aq. 0.9% sodium chloride solution. Then the SPE cartridge was washed with 5 mL of 0.9% aq. sodium chloride solution, which was collected in the product collection vial. The resulting solution was transferred through a sterile filter into the sterile 25 mL vial. The final total volume of the product solution was 15 mL (containing 10% ethanol, *v*/*v*).

#### Process validation

For the process validation, three consecutive batches of [^11^C]BMP were prepared and controlled. Validation runs were performed under the same operating conditions normally set for typical runs. As acceptance criteria a radioactivity concentration ≥ 100 MBq/mL and a final volume of 15 mL was set and each batch was analysed following the complete quality control program. The shelf life of the formulated product was limited to 120 min. Stability of the final radiotracer solution was assessed in the three consecutive batches of [^11^C]BMP. Radiochemical purity, pH and osmolality were additionally assessed after storage at room temperature at 60 min and 120 min after the end of synthesis (EOS).

### Quality control

Each batch of [^11^C]BMP was submitted to quality control, with the aim to evaluate chemical, radiochemical, radionuclidic and biological purity of the finished product. Failure to adhere to quality control specified limits required retesting of the batch. If the limits remained unmet, the final product could not be released, mandating rejection of the batch, as depicted in the flow chart provided in Additional file 1: Fig. S2. The used analytical methods were validated in three consecutive batches as recommended in the European Medicines Agency (EMA) “Guideline on the requirements to the chemical and pharmaceutical quality documentation concerning investigational medicinal products in clinical trials” (EMA 2022). The employed analytical procedures and product specifications were based on the current European Pharmacopoeia (Ph. Eur.) monographs “Extemporaneous preparation of radiopharmaceuticals (51,900)”, “Chemical precursors for radiopharmaceutical preparations (2902)”, “Radiopharmaceutical preparations (0125)”, “Raclopride ([^11^C]methoxy) injection (1924)”, “L-methionine ([^11^C]methyl) injection (1617)”, “Flumazenil (*N*-[^11^C]methyl) injection (1917)” and “Choline ([^11^C]methyl) injection (2462)”. Product specifications for [^11^C]BMP are listed in Table 1.

**Table 1:** Specifications and acceptance criteria for [^11^C]BMP based on a maximum administered volume of 15 mL (total batch volume)

Parameters	Test	Specification
Radioactivity concentration	Dose calibrator	≥ 100 MBq/mL
Identification	HPLC-radioactivity HPLC–UV	The principal peak in the radiochromatogram obtained with the test solution of [^11^C]BMP has a retention time ± 0.2 min of the principal peak in the chromatogram obtained with a reference solution of unlabelled BMP
Identification	Gamma spectrometry	The most prominent peak of the gamma spectrum is at 480–530 keV
Radiochemical purity	HPLC-radioactivity	[^11^C]BMP ≥ 95% 6-bromo-9-[^11^C]methylpurine ≤ 3% Other impurity ≤ 3%
Content of BMP	HPLC–UV	≤ 3.34 µg/mL
Content of 6-bromo-7H-purine	HPLC–UV	≤ 1 µg/mL
Radionuclidic purity*	Gamma spectrometry Dose calibrator	Peak 480–530 keV Half-life
Half-life	Dose calibrator	20.3 ± 2 min
Residual solvents	Gas chromatography	Acetonitrile ≤ 273 ppm THF ≤ 480 ppm
pH	pH meter	4.5–8.5
Osmolality	Osmometer	200–400 mosm/kg
Sterility*	Ph. Eur	Sterile
Bacterial endotoxins*	Ph. Eur	< 175 endotoxin units (EU) / maximum recommended dose (mL)

*The tests labelled with an asterisk were performed after the release of the radiopharmaceutical, as the test duration was not compatible with the radionuclide half-life of ^11^C (i.e., sterility test and radionuclidic purity) and/or for radiation protection reasons (i.e., bacterial endotoxins test)

For identification and determination of radiochemical purity of [^11^C]BMP and content of unlabelled BMP and 6-bromo-7H-purine, the analytical radio-HPLC system described in the General section was used (see Additional file 1: Fig. S3 for an analytical HPLC chromatogram of a mixture of 6-bromo-7H-purine, BMP and 6-bromo-9-methylpurine). Analysis specification for identification was that the main radioactive peak had the same retention time evidenced by the system suitability test for standard BMP ± 0.2 min. For radiochemical purity, the peak area for [^11^C]BMP had to be ≥ 95% of the total peak areas, whereas the peak area for 6-bromo-9-[^11^C]methylpurine (i.e. the undesired regioisomer) had to be ≤ 3% of the total peak areas. For determination of BMP and 6-bromo-7H-purine content, the peak areas of BMP and 6-bromo-7H-purine had to be not more than the corresponding peak areas obtained with the reference system suitability test solution (1 µg/mL of 6-bromo-7H-purine and 3.34 µg/mL of BMP in aq. 0.9% saline solution) corresponding to ≤ 50 µg of unlabelled BMP and ≤ 15 µg of 6-bromo-7H-purine in a maximum administered volume of 15 mL. Test and acceptance criteria for the validation of the method for the determination of BMP and 6-bromo-7H-purine content using HPLC are provided in the Additional file 1: Table S1. Sterility and endotoxin testing were performed after decay of the radioactivity using procedures described in the Ph. Eur.

### Toxicity study

An extended single-dose toxicity study was conducted at BSL BIOSERVICE Scientific Laboratories Munich GmbH (Planegg, Germany) under good laboratory practice (GLP) in male and female healthy Wistar rats, Crl: WI(Han) (Charles River, Sulzfeld, Germany) after single i.v. administration of unlabelled BMP at a dose of 2 mg/kg body weight following the “ICH guideline M3(R2) on non-clinical safety studies for the conduct of human clinical trials and marketing authorisation for pharmaceuticals” (EMA 2009). The study was conducted with two groups including one dose group (2 mg/kg) and one control group. The test item was administered once to all animals of the dose group via slow i.v. bolus injection (5 mL/kg). Animals of the control group were handled identically as the dose group but received sterile aq. sodium chloride solution (0.9%, *w*/*v*), the vehicle used in the study. The two groups comprised 30 male and 30 female rats. During the period of the study, the animals were observed precisely each day for signs of toxicity and mortality. Body weight and food consumption were measured weekly. 10 animals of each group and sex were sacrificed one day after administration and then examined macroscopically and histopathologically. The choice of organs for histopathological assessment (brain, colon, heart, kidneys, liver and lung) was guided by the dynamic whole-body PET data of [^11^C]BMP measured in female C57BL/6J mice (Zoufal et al. 2019). In order to allow a detection of possible delayed toxicity, 5 animals per group and sex were observed for a period of 13 days following administration and examined macroscopically and histopathologically thereafter. A full histopathological evaluation of the tissues was performed on 5 animals per sex of dose and control animals sacrificed one day after administration and on all animals sacrificed 13 days after administration.

### Dosimetry

The human dosimetry of [^11^C]BMP was estimated based on previously published dynamic whole-body PET data in six female C57BL/6J mice measured after i.v. bolus injection of 32 ± 10 MBq of [^11^C]BMP (Zoufal et al. 2019). The dynamic PET data comprised 25 time points from 2.4 s to 80 min post injection. The widely accepted “%-kg/g”- method (Blau 1975; Stabin 2008) was applied for the interspecies extrapolation of the biokinetics, in which the standardised uptake value (SUV) of animals and humans was assumed to be identical. The mean of the SUVs of all subjects was converted into the organs’ activity per administered activity for every time point by scaling with the respective organ masses of the International Commission on Radiological Protection (ICRP) reference phantoms (Menzel et al. 2009). For the calculation of the residence time [defined as the time-integrated activity coefficient in the standardised Medical Internal Radiation Dose (MIRD) nomenclature (Bolch et al. 2009)] the activity of the first time point was assumed as the value for immediate uptake with trapezoidal integration for all other measured time points. After the last time point of 80 min, the biological elimination was set to zero, that is the contribution to the residence time after 80 min was calculated only by the radioactive decay of ^11^C. Blood, brain, gallbladder contents, heart wall, kidneys, liver, lungs, and the contents of the small intestine as well as urinary bladder were used as source organs for the MIRD methodology (Loevinger et al. 1991; Stabin 2008), with the rest of the radiotracer homogeneously distributed in the remaining tissue. The IDAC software (Andersson et al. 2017) was applied for the computation of the organ absorbed doses and the effective dose as defined in the ICRP publication 103 (ICRP 2007).

## Results

### Automated radiosynthesis of [^11^C]BMP

**Fig. 2:**
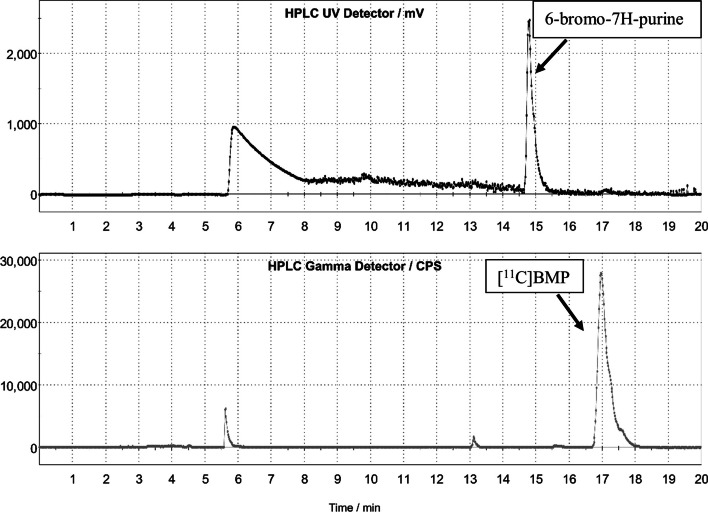
Representative reversed-phase semi-preparative HPLC chromatogram for the purification of [^11^C]BMP. The upper channel represents UV absorption at 254 nm and the lower channel radioactivity detection

[^11^C]BMP was prepared by regioselective *N*^7^-[^11^C]methylation of 6-bromo-7H-purine by [^11^C]CH_3_OTf in presence of TMP·MgCl (Fig. 1). The radiosynthesis was automated in a TRACERlab™ FX2 C synthesis module, by means of which [^11^C]BMP was purified after the radiolabelling reaction with reversed-phase semi-preparative HPLC. A representative semi-preparative HPLC chromatogram for the purification of [^11^C]BMP is shown in Fig. 2. For removal of HPLC solvent, the product fraction was diluted with water and passed over a SPE cartridge on which the product was retained. After washing the cartridge with water, [^11^C]BMP was eluted with ethanol. The ethanol was diluted with 0.9% aq. sodium chloride solution. The final formulation was thus very simple and 0.9% aq. sodium chloride solution and ethanol were the only excipients included in the product vial. The finished product was considered as a single-dose preparation. Stability tests demonstrated that the composition of the [^11^C]BMP solution did not undergo significant changes in chemical and radiochemical purity during the pre-defined shelf life of the formulation (120 min). At 120 min, the radiochemical purity of all tested batches was better than the required limit of 95% and no degradation products nor any changes in the corresponding UV area of BMP were observed. The first three batches validated the robustness of the procedure, which could further be demonstrated with 29 consecutive radiosynthesis batches, part of which were used for administration to healthy humans. Starting from 52 ± 4 GBq of [^11^C]CH_3_I, 3.7 ± 0.8 GBq of [^11^C]BMP was obtained in a decay-corrected radiochemical yield of 20.5 ± 5.2%, based on [^11^C]CH_3_I (*n* = 28), in a total synthesis time of approximately 43 min. The radiochemical purity of [^11^C]BMP was greater than 99% and molar activity at EOS was 197 ± 130 GBq/μmol. The content of unlabelled BMP of all batches was better than the defined specification of 3.34 µg/mL (0.52 ± 0.55 µg/mL). The content of 6-bromo-7H-purine was below the limit of quantification (0.615 µg/mL). All batches prepared were sterile with endotoxin levels below 175 EU/15 mL. No deviations in pH and osmolality were observed.

### Toxicity study

An extended single-dose toxicity study conducted after single i.v. administration of unlabelled BMP at a dose of 2 mg/kg showed that BMP was well tolerated with no test item-related effect observed on mortality, clinical signs, body weight development, food consumption, haematology, blood coagulation, clinical biochemistry, urinalysis, gross pathological findings, organ weight and histopathology in male and female rats sacrificed at the end of the study period.

### Dosimetry

The human dosimetry estimates provided an effective dose of 4.70 ± 1.68 μSv/MBq and 4.43 ± 1.58 μSv/MBq for male and female subjects, respectively. The organ absorbed doses did not show any conspicuous values (Additional file 1: Table S2). The highest organ absorbed doses were estimated for the urinary bladder wall, which was consistent with a predominantly urinary excretion of [^11^C]BMP-derived radioactivity (Zoufal et al. 2019).

## Discussion

To further improve the published radiosynthesis approaches of [^11^C]BMP (Okamura et al. 2024, 2009b; Zoufal et al. 2019), we developed a regioselective *N*^7^-[^11^C]methylation procedure of 6-bromo-7H-purine with [^11^C]CH_3_OTf in presence of TMP·MgCl (Fig. 1). Our radiosynthesis conditions were based on the work by Chen et al., who tested various bases to achieve selectivity for *N*-alkylation at the sterically more hindered position of various 1,3-azoles (Chen et al. 2016). These authors identified the Hauser base TMP·MgCl as a base which provided good selectivity, high yields and which was compatible with the presence of various sensitive functional groups. The selectivity of the *N*-methylation was shown to be determined by the steric hindrance of the site of methylation by the adjacent substituent (6-bromo in the case of 6-bromo-7H-purine) (Chen et al. 2016). To the best of our knowledge, regioselective *N*-alkylation has not yet been utilised in the synthesis of PET radiotracers. The use of TMP·MgCl in the synthesis of [^11^C]BMP proved to be a valuable method for protective-group-free radiolabelling and might also enable the regioselective radiolabelling of other 1,3-azoles.

The radiolabelling precursor 6-bromo-7H-purine used in the radiosynthesis of [^11^C]BMP was manufactured under GMP conditions, which is a current requirement of the Austrian regulatory authority because “in-house preparations” of radiopharmaceuticals for immediate use do not need to be performed under GMP conditions as per Austrian national regulations. It should be noted in this context that the new Clinical Trial Regulation 536/2014, which entered into force in the beginning of 2023, exempts “in-house prepared” diagnostic radiopharmaceuticals used in clinical trials from the GMP requirement (Peñuelas et al. 2019).

Among the 29 syntheses conducted, one synthesis failed due to the unexpected formation of the 9-isomer (see Additional file 1: Fig. S4). The synthesis failure may be attributed to a potential issue with the filling of the Hauser base vial, resulting in an unfavourable base-to-precursor ratio. However, all other batches met all quality control criteria (Table 1) and were suitable for human application.

Although the reversed-phase semi-preparative HPLC system had some limitations, such as the requirement of a step gradient and occasional peak splitting, it consistently achieved good separation between radiolabelling precursor and product in all syntheses (Fig. 2). The separation resolution between the 7- and 9-isomers is not critically important, as the formation of the 9-isomer was only observed once and was easily identifiable in the analytical HPLC system (Additional file 1: Figs. S3 and S4). The decision to employ this harsh step gradient stems from the significant influence of THF, which complicates the separation of radiolabelling precursor and product when starting with a higher acetonitrile percentage. Ideally, a continuous gradient would be preferred, but this was not feasible with the stock configuration of the employed synthesis module. The use of a reversed-phase HPLC system enabled a straightforward removal of HPLC solvent by means of SPE and obliviated the need for evaporation of HPLC solvent, as in the previously published radiosyntheses of [^11^C]BMP (Okamura et al. 2009b, 2024; Zoufal et al. 2019). Our decay-corrected radiochemical yield of [^11^C]BMP (20.5 ± 5.2%, based on [^11^C]CH_3_I) was lower than the radiochemical yield reported by Okamura et al. (22–35%, based on [^11^C]CO_2_) (Okamura et al. 2024), but the final amount of [^11^C]BMP obtained at EOS by our radiosynthesis (3.7 ± 0.8 GBq) was largely sufficient for human application.

BMP is a new chemical entity which has never been administered to humans before. In the clinical study, [^11^C]BMP is administered at a maximum of two single i.v. injections, with the total amount of drug substance from both administrations being less than 100 µg, meeting the definition of a microdose as per the "ICH guideline M3(R2) on non-clinical safety studies for the conduct of human clinical trials and marketing authorisation for pharmaceuticals" (EMA 2009). We therefore conducted an extended single-dose toxicity study in male and female rats following approach 1 in the ICH guideline M3(R2) (EMA 2009). The precondition for approach 1 is that the total administered dose in the clinical study is ≤ 100 µg and ≤ 100th of the no observed adverse effect level (NOAEL) in animal toxicity studies and ≤ 100th of the pharmacologically active dose. We selected for the toxicity study a dose of 2 mg/kg body weight, which was 1198-times higher than the intended maximum dose for the clinical PET study (0.1 mg/60 kg = 0.00167 mg/kg). No adverse effects related to BMP were observed and the NOAEL is therefore greater than 2 mg/kg. Consequently, treatment with the intended dose of ≤ 100 µg is not anticipated to pose any health risks to human subjects and [^11^C]BMP is expected to be safe and well tolerated in humans. Based on the results of the toxicity study, the specification for content of unlabelled BMP was set to ≤ 3.34 µg/mL (Table 1) corresponding to a total amount of ≤ 50 µg per administered dose of [^11^C]BMP with a maximum of two administrations per subject. For the radiolabelling precursor 6-bromo-7H-purine, the acceptance limit was set to ≤ 1 µg/mL (Table 1) in consideration of literature data in which a dose of 100 mg/kg (intraperitoneal) of 6-bromo-7H-purine exerted no toxicity in mice (Sartorelli et al. 1962).

For a clinical trial application with a new radiopharmaceutical, dosimetry estimates, based on biodistribution data in animals, are required to predict the radioactive exposure in humans (EMA 2018). It should be noted that there is an ongoing debate whether dosimetry estimates are indeed necessary for ^11^C-labelled radiotracers as reported effective doses for different ^11^C-radiotracers show a low degree of variability (Zanotti-Fregonara et al. 2021). We estimated human dosimetry based on previously published dynamic whole-body PET data acquired in 6 female C57BL/6J mice (Zoufal et al. 2019). The herein reported dosimetry data (Additional file 1: Table S2) showed a maximum effective dose of 4.70 ± 1.68 µSv/MBq for male and 4.43 ± 1.58 µSv/MBq for female subjects, which is in the typical range of ^11^C-tracers (average effective dose of 77 different ^11^C-tracers: 5.2 ± 1.7 µSv/MBq) (Zanotti-Fregonara et al. 2021). Therefore, an injected activity amount of 400 MBq of [^11^C]BMP is expected to result in an effective dose of 1.88 mSv for men and 1.77 mSv for women, which is well below the limit of 10 mSv for studies in healthy volunteers aged less than 50 years (ICRP 1992). The radiochemistry validation, toxicity and dosimetry data provided here were used to prepare an IMPD that was included in a clinical trial application which was approved by the Ethics Committee of the Medical University of Vienna and the Austrian Agency for Health and Food Safety (EUDRACT Nr 2021-006348-29).

## Conclusions

In conclusion, [^11^C]BMP as a radiotracer for in vivo imaging of MRP1 function, can be produced in sufficient quantities and excellent quality using a novel regioselective radiosynthesis method, setting the stage for an ongoing clinical trial. Toxicity evaluations indicate no adverse effects, underlining the favourable safety profile of [^11^C]BMP and indicating its promising potential for human application.

### Supplementary Information

**Additional file1**. **Figure S1**: Flow chart of the TRACERlab™ FX2 C synthesis module; **Figure S2**: Flow chart for the preparation process of [^11^C]BMP; **Figure S3**: Analytical HPLC chromatogram of a mixture of 6-bromo-7H-purine, BMP and 6-bromo-9-methylpurine; **Figure S4**: Analytical HPLC chromatogram of the single failed batch; **Table S1**: Test and acceptance criteria for the validation of the method for the determination of BMP and 6-bromo-7H-purine content; **Table S2**: Estimated human absorbed organ doses and effective dose of [^11^C]BMP.

## Data Availability

The datasets used and/or analysed in this work are available from the corresponding author on reasonable request.

## Abbreviations

BMP: 6-Bromo-7-methylpurine
[^11^C]BMP: 6-Bromo-7-[^11^C]methylpurine
^11^C: Carbon-11
[^11^C]CO_2_: [^11^C]Carbon dioxide
EMA: European medicines agency
EOS: End of synthesis
EU: Endotoxin unit
GLP: Good laboratory practice
GMP: Good manufacturing practice
HPLC: High-performance liquid chromatography
ICRP: International Commission on Radiological Protection
IMPD: Investigational Medicinal Product Dossier
i.v.: Intravenous
[^11^C]CH_4_: [^11^C]Methane
[^11^C]CH_3_I: [^11^C]Methyl iodide
[^11^C]CH_3_OTf: [^11^C]Methyl triflate
MRP1: Multidrug resistance-associated protein 1
NMR: Nuclear magnetic resonance
NOAEL: No observed adverse effect level
PET: Positron emission tomography
Ph. Eur.: European Pharmacopoeia
SPE: Solid-phase extraction
SUV: Standardised uptake value
THF: Tetrahydrofuran
TMP·MgCl: 2,2,6,6-Tetramethylpiperidine magnesium chloride
UV: Ultraviolet
