# Supplementary material for: Advancing 6-bromo-7-[11C]methylpurine to clinical use: improved regioselective radiosynthesis, non-clinical toxicity data and human dosimetry estimates
Source: EJNMMI Radiopharm Chem. 2024 Apr 29;9:34. doi: 10.1186/s41181-024-00265-z (PMC11058743; doi:10.1186/s41181-024-00265-z)
Supplement: Supplementary file 1 — Additional file1. Figure S1: Flow chart of the TRACERlab™ FX2 C synthesis module; Figure S2: Flow chart for the preparation process of [11C]BMP; Figure S3: Analytical HPLC chromatogram of a mixture of 6-bromo-7H-purine, BMP and 6-bromo-9-methylpurine; Figure S4: Analytical HPLC chromatogram of the single failed batch; Table S1: Test and acceptance criteria for the validation of the method for the determination of BMP and 6-bromo-7H-purine content; Table S2: Estimated human absorbed organ doses and effective dose of [11C]BMP. [file 41181_2024_265_MOESM1_ESM.docx]

**Supplementary material**

reaction vessel

product collection vial

SPE cartridge

Vials 1-6

bulb

Fig S1 Flow chart of the TRACERlab™ FX2 C synthesis module


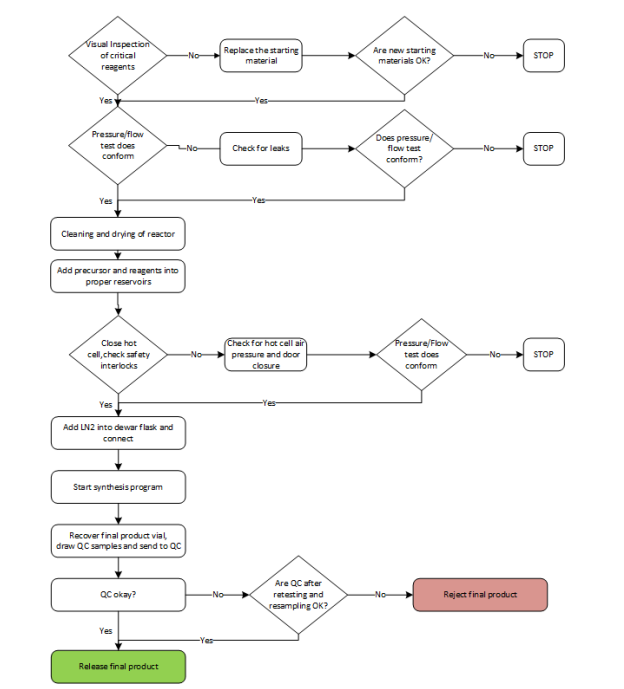


Fig S2 Flow chart for the preparation process of [^11^C]BMP

6-bromo-7H-purine

6-bromo-9-methylpurine

BMP

Fig S3 Analytical HPLC chromatogram of a mixture of 6-bromo-7H-purine (0.625 µg/mL), BMP (1.25 µg/mL) and 6-bromo-9-methylpurine (1.25 µg/mL).

[^11^C]BMP

6-bromo-9-[^11^C]methylpurine

Fig S4 Analytical HPLC chromatogram of the single failed batch, showing a clear separation of [^11^C]BMP from the impurity 6-bromo-9-[^11^C]methylpurine. The upper channel represents radioactivity detection and the lower channel UV absorption at 274 nm.

Table S1 Test and acceptance criteria for the validation of the method for the determination of BMP and 6-bromo-7H-purine content using HPLC

| **Test** | **Acceptance criteria** | **Values** |
| --- | --- | --- |
| **Specificity** | ≥ 1.5 | 6-bromo-7H-purine/BMP 4.45  BMP/6-bromo-9-methylpurine 3.42 |
| **Repeatability** | CV ≤ 5% | ≤ 5% |
| **Linearity** | r^2^ ≥ 0.99 | r² = 0.99999 |
| **Limit of quantification (LOQ)** | CV ≤ 5% for standard BMP  CV ≤ 5% for impurity | BMP 1.25 µg/mL CV 0.29%  6-bromo-7H-purine 0.615 µg/mL CV 2.86% |

CV, coefficient of variation; r, Pearson correlation coefficient

Table S2 Estimated human absorbed organ doses and effective dose based on dynamic PET data in female C57BL/6J mice after i.v. injection of [^11^C]BMP

| **Organs** | **Adult Male (µGy/MBq)** | **Adult Female (µGy/MBq)** |
| --- | --- | --- |
| Adipose/residual tissue | 2.60 | 2.61 |
| Adrenals | 3.35 | 3.78 |
| Alveolar-interstitial | 4.00 | 4.57 |
| Brain | 3.15 | 4.19 |
| Breast | 1.95 | 2.19 |
| Bronchi bound | 2.72 | 3.03 |
| Bronchi sequestered | 2.72 | 3.03 |
| Bronchioles | 4.00 | 4.50 |
| Colon wall | 3.04 | 4.13 |
| Endosteum (bone surface) | 3.39 | 2.92 |
| ET region | 3.75 | 1.57 |
| ET1 basal cells | 2.07 | 1.23 |
| ET2 basal cells | 3.75 | 1.57 |
| Eye lenses | 3.29 | 1.64 |
| Gallbladder wall | 5.03 | 6.21 |
| Heart wall | 2.94 | 3.40 |
| Kidneys | 7.55 | 9.38 |
| Left colon wall | 2.22 | 3.39 |
| Liver | 3.91 | 4.41 |
| Lung | 3.57 | 4.03 |
| Lymphatic nodes | 3.20 | 3.40 |
| Lymph nodes in ET region | 1.71 | 1.39 |
| Lymph nodes in sys | 3.49 | 3.75 |
| Lymph nodes in thoracic region | 1.61 | 1.78 |
| Muscle | 2.44 | 2.76 |
| Oesophagus | 2.28 | 2.39 |
| Oral mucosa | 3.30 | 2.17 |
| Ovaries | - | 7.52 |
| Pancreas | 3.13 | 3.56 |
| Pituitary gland | 13.5 | 3.02 |
| Prostate | 9.38 | - |
| Recto-sigmoid colon wall | 5.77 | 7.52 |
| Red (active) bone marrow | 3.49 | 3.31 |
| Right colon wall | 2.49 | 3.16 |
| Salivary glands | 3.15 | 2.23 |
| Skin | 1.84 | 1.92 |
| Small intestine wall | 4.05 | 5.60 |
| Spleen | 2.26 | 2.52 |
| Stomach wall | 2.19 | 2.54 |
| Testes | 2.45 | - |
| Thymus | 2.31 | 2.50 |
| Thyroid | 2.22 | 2.23 |
| Tongue | 2.62 | 1.39 |
| Tonsils | 5.54 | 2.31 |
| Ureters | 4.48 | 5.24 |
| Urinary bladder wall | 26.3 | 34.7 |
| Uterus/cervix | - | 12.2 |
| **Effective dose (µSv/MBq)** | **4.70 ± 1.68** | **4.43 ± 1.58** |
